# “I had to change my attitude”: narratives of most significant change explore the experience of universal home visits to pregnant women and their spouses in Bauchi State, Nigeria

**DOI:** 10.1186/s13690-021-00735-9

**Published:** 2021-11-18

**Authors:** Loubna Belaid, Umaira Ansari, Khalid Omer, Yagana Gidado, Muhammed Chadi Baba, Lois Ezekiel Daniel, Neil Andersson, Anne Cockcroft

**Affiliations:** 1 Department of Family Medicine, McGill University, Participatory Research at McGill (PRAM), 5858 Cote des Neiges, suite 300, Montreal, Quebec, Canada; 2 Centro de Investigación de Enfermedades Tropicales (CIET), Universidad Autónoma de Guerrero, Acapulco, Mexico; 3 Federation of Muslim Women Association of Nigeria (FOMWAN), Bauchi Chapter, Bauchi, Nigeria; 4 Bauchi State Primary Health Care Development Agency, Bauchi, Nigeria

**Keywords:** Behavior change, Maternal health, Child health, Narratives, Home visits, Thematic analysis, Male involvement

## Abstract

**Background:**

Universal home visits to pregnant women and their spouses in Bauchi State, northern Nigeria, discussed local evidence about maternal and child health risks actionable by households. The expected results chain for improved health behaviours resulting from the visits was based on the CASCADA model, which includes Conscious knowledge, Attitudes, Subjective norms, intention to Change, Agency to change, Discussion of options, and Action to change. Previous quantitative analysis confirmed the impact of the visits on maternal and child outcomes. To explore the mechanisms of the quantitative improvements, we analysed participants’ narratives of changes in their lives they attributed to the visits.

**Methods:**

Local researchers collected stories of change from 23 women and 21 men in households who had received home visits, from eight male and eight female home visitors, and from four government officers attached to the home visits program. We used a deductive thematic analysis based on the CASCADA results chain to analyze stories from women and men in households, and an inductive thematic approach to analyze stories from home visitors and government officials.

**Results:**

The stories from the visited women and men illustrated all steps in the CASCADA results chain. Almost all stories described increases in knowledge. Stories also described marked changes in attitudes and positive deviations from harmful subjective norms. Most stories recounted a change in behaviour attributed to the home visits, and many went on to mention a beneficial outcome of the behaviour change. Men, as well as women, described significant changes. The home visitors’ stories described increases in knowledge, increased self-confidence and status in the community, and, among women, financial empowerment.

**Conclusions:**

The narratives of change gave insights into likely mechanisms of impact of the home visits, at least in the Bauchi setting. The compatibility of our findings with the CASCADA results chain supports the use of this model in designing and analysing similar interventions in other settings. The indication that the home visits changed male engagement has broader relevance and contributes to the ongoing debate about how to increase male involvement in reproductive health.

## Background

Maternal mortality in Nigeria remains among the highest in the world, with an estimated national Maternal Mortality Ratio (MMR) of 814 per 100,000 live births [1]; mortality is even higher in the north of the country [2]. Nigeria also has high rates of under-5 years mortality and infant mortality, especially in the north [3]. International recommendations for improving maternal and newborn health focus on facility-based care [4]. But in Nigeria the quality of maternal and child health services from over-stretched and under-resourced facilities is often too low to be effective [5–8]. A systematic review of qualitative studies reported that perceived poor quality and disrespectful services from health facilities were important reasons for women avoiding giving birth in facilities in low- and middle-income countries [9].

Factors like poverty and lack of education that increase maternal and child health risks also limit women’s ability to attend facility-based services [10]. Community-based interventions could help to overcome this problem, and several systematic reviews have concluded that a range of community-based interventions, including home visits, can improve maternal, newborn, and child health outcomes in low- and middle-income countries [11–19]. Most of the reviews reported primarily on ultimate outcomes of maternal and neonatal mortality and morbidity [11, 13–17, 19]. Many considered intermediate outcomes likely to be related to these outcomes, such as improved neonatal care practices [11, 12], improved care seeking for early childhood illnesses [15], and reductions in socioeconomic inequalities in care [18]. Some reported small increases in antenatal care [17, 19] or in facility-based births [14].

There are some important gaps in knowledge, acknowledged in the reviews. The included studies of community-based interventions did not examine changes in factors like beliefs, self-efficacy, and perceived social norms, although these are likely to mediate behaviour change in response to the interventions [20]. Most of the reviews included a range of interventions, not only home visits [13–15, 17–19]. Some of the reviews included only studies from South Asia [11, 16, 17] and the great majority of the included studies in all the reviews were from South Asia, with few from Africa and even fewer from West Africa.

The reviews of community-based interventions generally did not consider the role of male involvement in improving maternal and child health outcomes, although a review of community mobilization in rural South Asia suggested that including male mobilizers might help [17] and a recent review of health education packages for maternal and child health noted that some studies included spouses and other family members [19]. Systematic reviews of studies in low- and middle-income countries have concluded that male involvement is associated with increased use of maternal care services, such as attendance of women for antenatal care, institutional delivery, skilled birth attendance, and post-natal visits [21–23]. Few studies of interventions to engage men in maternal and newborn health have directly measured outcomes of knowledge, attitudes or behaviour among men [24–27].

Our study addresses some of these knowledge gaps by exploring the potential mechanisms underlying the quantitative impact on maternal and child health outcomes of a home visits program in northern Nigeria. A cluster randomized controlled trial in Bauchi State, Nigeria, aimed to measure the impact of universal home visits to pregnant women and their spouses on maternal and infant outcomes and on male knowledge and attitudes [28]. Female home visitors visited all pregnant women every 2 months during their pregnancies and again after delivery, and male visitors visited their husbands. The visitors shared the same evidence with both women and their spouses about actionable factors related to maternal and infant health. For maternal health, they shared evidence from a study in Bauchi, which found four actionable factors related to maternal morbidity: heavy work during pregnancy, the experience of domestic violence during pregnancy, lack of spousal communication about pregnancy and delivery, and lack of knowledge about danger signs during pregnancy and delivery [29]. For child health, they shared evidence about the benefits of childhood immunizations, about breastfeeding (including use of colostrum), and about prevention and management of childhood diarrhoea, including information from surveys in Bauchi [30]. Quantitative analysis of questionnaire responses in the trial revealed significant impacts of the home visits intervention: reduced complications of pregnancy and delivery and improved targeted risk factors [31]; improvements in child health outcomes [32]; and improved male knowledge and behaviour [33].

To supplement our quantitative analysis of the impact of the home visits program, we conducted a qualitative study to explore the mechanisms underlying the quantitative impact. For our qualitative study, we used a narrative approach to explore how participants felt the program affected them. We collected and analysed stories of change from visited pregnant women and visited spouses of pregnant women, from the home visitors themselves, and from government officers attached to the program.

## Methods

### Context

The home visits program took place in Toro Local Government Area (LGA) in Bauchi State, in north-eastern Nigeria. Most of the five million residents in Bauchi are Muslim, polygamy is common and family sizes are large. About three-quarters of women in Bauchi have no education, compared with 38% nationally [3]. In a 2013 survey, 61% of women in Toro LGA reported four or more antenatal care visits in a government health facility in their last pregnancy; 27% had a skilled attendant for their last delivery; 60% did not reduce heavy work before the last trimester of pregnancy; and 16% experienced intimate partner violence during their last pregnancy [30]. Only 6% of children aged 12–23 months in Bauchi received all basic vaccinations; 26% of Bauchi children under 5 years old had diarrhoea in the last 2 weeks; 51% of Bauchi children under 5 years old were stunted and 24% wasted [3]. Bauchi State Primary Health Care Development Agency is responsible for primary health care in the State. The agency actively collaborated in the trial of the home visits program.

### Narrative evaluation

We drew on the Most Significant Change technique (MSC) to collect narratives of change from visited women and their spouses, from female and male home visitors, and from collaborating government officers. MSC captures and collates stories about program effects from participants. It can identify both intended and unexpected outcomes, and it provides insight into how an intervention effects those involved [34]. Although MSC is widely used in evaluation of development projects it has been much less used in the evaluation of health interventions. A recent report summarized health sector experience with using MSC in participatory initiatives [35].

### Sampling

In May 2017, the research team asked home visitors to identify women and men “who might have a story to tell” about their experience of the program, based on the home visitors’ knowledge of community members who were more vocal and communicative, or who had already shared their experiences during the home visits. In a meeting with male and female home visitors, the research team invited them to come forward if they were interested in sharing a story about their experience of working in the program. The local research team nominated several government officers attached to the program who might have a story to tell.

### Story collection

After days of training in the collection of stories, four members of the local research team who were field coordinators for the home visits program (two women and two men, aged from 32 to 43 years old) collected stories from visited women and men from different households, and from home visitors. Women collected the stories from women, and men collected the stories from men. The field coordinators are all graduates with several years of experience collecting qualitative data. One of the authors, a researcher experienced with the most significant change technique (UA), collected stories from government officers attached to the home visits program.

After verbal consent, the coordinators asked the household respondents “Please tell me a story describing what you think is the most significant change in your life since we started visiting you in the home visits program. Your story could be about a positive change in your life, or about a negative change.” For the home visitors and government officers, the question was the same, but about change “since you started working in the home visits program”. The data collection form included suggested probes to help respondents elaborate on their stories. The coordinators collected the stories in Hausa and then translated them into English; UA collected stories from the government officers in English.

The coordinators met household storytellers in or near their households and they shared their stories in a quiet, private place. The home visitors who wanted to tell a story stayed after the recruitment meeting and told their story in private. UA collected stories from government officers in private, either in their offices or during a field visit.

### Analysis

The home visits program is based on the idea that sharing evidence with women and men in households can empower them to tackle upstream risk factors for maternal and child health. It makes use of the CASCADA results chain for behaviour change [36, 37], a version of the Theory of Planned Behaviour [38]. CASCADA is an acronym for a partial order of intermediate outcomes between knowledge and action: Conscious knowledge, Attitudes, Subjective norms, intention to Change, Agency to make the change, Discussion with family, peers and neighbours, and finally Action. We have found the CASCADA results chain useful in resource-poor settings to guide design of interventions [39, 40] and as a framework for qualitative and quantitative analysis of intervention effects [41, 42].

A deductive thematic analysis used the steps in the CASCADA results chain as themes to analyze the stories from the visited women and men in households. We followed Braun and Clark’s guide for thematic analysis [43]. The guide consists of six steps: (i) familiarising with the data, (ii) generating initial codes, (iii) searching for themes, (iv) reviewing themes, (v) defining and naming themes (vi) producing a report. LB, UA and AC read all stories, identified themes and clustered themes that shared the same ideas. In a member checking exercise, the local research team, including supervisors who collected stories, checked and refined the initial list of themes. Further analysis organized the themes into categories and subcategories to identify meanings and patterns.

We used an inductive thematic approach to analyse the stories from the home visitors and government officials; we did not expect the program to change their behaviours or perceptions, but we were interested in hearing from them how their participation impacted their lives.

### Trustworthiness

We used strategies based on the criteria of Guba to ensure the trustworthiness of the study [44, 45]. We used validated methods to collect stories (Most Significant Change technique) and to analyse the data (thematic analysis). We triangulated the findings by data sources and gender (females and males from visited households, female and male home visitors, and female and male government officials). We carried out member checking exercises with storytellers and with the local research team. To increase transferability, we describe the participants and context of the study. We increased conformability through debriefing sessions in which the research team members discussed their own biases, assumptions, beliefs, and suppositions that might affect their interpretation of findings.

## Results

We reached saturation throughout the stages of the research [46]. In data collection, saturation was when the collected data became repeated: after 64 stories (23 from visited women, 21 from visited men, 16 from home visitors, and four from government officials) we did not identify new data. In data analysis, we reached saturation when there were no new themes emerging from the stories from home visitors and government officers. For analysis of the stories from women and men in households, the pre-determined themes from our CASCADA framework adequately reflected the data.

### Stories from the visited women and men

Twenty three visited women and 21 visited men from different households gave a story. None of the women and men approached declined to tell a story. The mean age of the visited women was 32 years (20–45 years). All were married, with a range of three to eight children. Many had no education; none had more than primary education. Most of them reported their occupation as taking care of the household and children. A few were involved in small businesses, mostly from within the home. The mean age of the visited men was 40.3 years (25–62 years). All were married, with from one to 14 children, and with either primary or secondary education. Twelve of the men had one wife, the others had two or three wives. Their occupations included teaching, health work, petty trade, small business ownership, and farming.

#### Conscious knowledge

All the storytellers mentioned that the visits had increased their knowledge. They specifically reported increases in knowledge about the factors related to maternal and child health that were covered in the visits.

*“She [the home visitor] explained to me about the different kinds of immunization for different types of childhood illnesses. This was all new to me. I realized that I had been depriving my children of maximum health care and protection.” (woman, 28 years old, with four children, from a rural community).*

*“I came to understand that the headaches my [pregnant] wife was suffering from were actually a danger sign. She needed medical help.” (man, 27 years old, with two children, from urban community).*

#### Attitudes

Many of the storytellers described previous unhelpful attitudes in themselves or their spouses, especially about childhood immunization and heavy work during pregnancy. They attributed changes in these attitudes to the discussions with the home visitors. It was clear that the visited women and men believed the evidence the home visitors presented to them.

*“I believed that immunization was a waste of time, and it is unnecessary for a child. After all, other children who are not immunized still grow up and become good citizens …*. *I was two months pregnant with my last child when your worker started visiting me. She told me that immunization is very vital for a child’s health. I gradually realized that a child who receives immunization is immune to childhood illnesses and suffers less during an outbreak of childhood illnesses.” (woman, 28 years old, with four children, from a rural community).*

*“I was forced into thinking that I had to change my attitude. I became friendly to my family.” (man, 58 years old, with 12 children, from a rural community).*

#### Subjective norms

For several of the topics addressed in the home visits, there are widespread beliefs in Bauchi about health behaviours, often passed down within families, and sometimes unhelpful. Stories suggested that the visits had led men and women to reflect on these beliefs and had enabled them to deviate from unhelpful norms.

*“When I got pregnant, I was told by my family members that heavy work is good for pregnant women because it helps them to be strong and healthy during delivery. Last year, I was six months pregnant with my sixth child... your field worker started visiting my house … she explained the dangers of doing heavy work. …. I would not have been able to reduce heavy work if your worker had not told me to do it.” (woman, 28 years old, with six children, from an urban community).*

*“I was taught that a man could only be respected in his house if he is harsh … I believed that I could only be respected as a husband and as the head of the household if I was harsh in the house. I believed that domestic violence was a normal occurrence between a husband and his wife. When the home worker started visiting me, I understood that domestic violence is not good for a pregnant woman.” (man, 60 years old, from a rural community).*

#### Intention to change

Most of the storytellers described changes they had already made, rather than actions they intended to take. A few explained their intention to apply their new knowledge in the future.

*“I am now six months pregnant. I have decided to immunize my child after I give birth” (woman, 35 years old, with five children, from a rural community).*

Some stories used words showing how the intention to change had been part of the sequence leading to the action they described.

*“When I reached my fifth month of pregnancy, I decided to reduce my heavy work. I reduced my commercial washing.” (woman, 40 years old, with five children, from a rural community).*

#### Agency

Some stories described increased agency related to specific issues.

*“This programme has supported me on how to keep, manage and protect my wife’s pregnancy if she finds herself in any kind of danger during either pregnancy or the childbirth.” (man, 30 years old, with five children, from a rural community).*

Other storytellers described a feeling of confidence inspired by the home visits.

*“I felt very confident up to the time of my delivery … I had never experienced such an enjoyable pregnancy like my sixth pregnancy” (woman, 30 years old, with six children, from a rural community).*

Some men and women described feeling confident to tell others what they had found out from the home visitors.

*“I feel happy and ready to advise and inform everyone about the importance of immunization for children.” (man, 26 years old, with one child, from an urban community).*

## Discussion

The home visitors shared evidence with pregnant women and their spouses about the link between lack of communication between spouses and maternal health. Some storytellers described how they now discussed with their spouses about pregnancy, delivery, and child health.

*“I started discussing and sharing ideas with my wife regarding the issues of her pregnancy and childbirth. My life has changed as a result of this project, and especially my ability to speak with my wife and other family members*.” (man, 47 years old, with 14 children, from a rural community).

### Actions

Nearly all the stories included a description of actions the storytellers had taken as a result of their interaction with the home visitors.

*“I decided to reduce specific types of heavy work, like rice and corn processing. I have stopped pounding, fetching water, and collecting firewood” (woman, 30 years old, with four children, from a rural community).*

Many storytellers explained that the actions they had taken as a result of the home visits had led to tangible benefits, and this reinforced their conviction that they had made the right changes.

*“This knowledge led me to stop her from engaging in any form of heavy work. I finally received the benefit of this knowledge when my wife delivered successfully without any complications or problems. She delivered at home. She did not need to go to hospital. Both the mother and the child are in good health.” (man, 30 years old, with 10 children, from a rural community).*

### Stories from the home visitors

We collected stories from 16 home visitors (eight women and eight men). The mean age of the home visitors was 27. 4 years (range 20–37 years), and they all had either primary or secondary level education. They described marked changes in their lives that they attributed to their work as home visitors in the program, with some differences between men and women in the focus of their stories.

#### Financial empowerment and independence

For most of the female home visitors, this was the most important change they experienced. The stipend they received for their work as home visitors, although quite small, enabled them to respond to their families’ financial needs:

*“This organization [the home visit program] has empowered me greatly. I am able to send my children to school. This is a great privilege for me. I also help my husband to meet the needs of our family.” (Female home visitor, 37 years old, married with six children).*

None of the male home visitors considered the financial remuneration they got from the home visiting work as the most significant change in their lives.

#### Recognition within their communities

For many male home visitors, the most important change in their lives was that their participation in the program increased their social recognition within their communities:

*“I have gained respect and recognition by my community. Wherever I go, people recognize me and want to talk to me. They call me ‘doctor’ and respect me tremendously. I will work as a home visitor even if I do not get paid for my work. The respect and recognition I have earned here has changed my life.” (Male home visitor, 25 years old).*

#### Improved self-confidence and communication skills

Some men described how their work as home visitors had improved their self-confidence and communication skills, beyond the specific topic of home visits.

*“When I started working on this program, I was afraid of speaking with married men. I soon overcame my shyness. I now feel confident and enlightened on how to speak publicly.” (Male home visitor, 21 years old).*

This topic did not feature in stories from female home visitors.

#### Increased knowledge about maternal and child health

Both male and female home visitors described how their participation in the program had improved their knowledge about maternal and child health. They felt confident to disseminate this knowledge in the households they visited.

*“The training I received from this organisation has helped me understand these danger signs during pregnancy and childbirth … I am now able to educate women who are less privileged to also know about these danger signs.” (Female home visitor, 31 years old, with three children).*

### Stories from the government officers

The four government officers (three men and one woman) who told stories were aged from 30 to 53 years. One held a position in a State -level government college, and the other three held positions within the Toro LGA health sector. All of them told stories about how the program had impacted their work. One described improved experiential understanding of communities’ needs as a result of involvement with the program.

*“As a result of my interactions with communities, I soon realized that I was better able to interact with people who came to see me at the health facility. I was able to solve their problems because now I was familiar with their issues” (Female government officer, 49 years old).*

Another described the benefit of receiving timely quantitative data from household level, which helped him plan effectively.

*“When we started the home visit program, I soon began receiving summary sheets of household data. I was able to calculate the number of pregnant women in our communities just by a glance. I appreciate that we have information about what is happening in our communities. This was an important change to how I used to do my work previously.” (Male government officer, 53 years old).*

## Discussion

### Stories from women and men who had experienced home visits

The stories from the women and men who had experienced home visits reflected all the steps in the CASCADA results chain. All respondents mentioned an increase in *knowledge*, usually as the first part of their story, but stories also reflected changing attitudes, positive deviation from harmful subjective norms, intention to change, increased agency, discussion of new knowledge, and behaviour change. This is further evidence for the relevance of the CASCADA model in designing and analysing public health interventions [39–42].

Simply giving people information about health risks is often not effective in changing health behaviours [47]. We believe the success of the home visits in leading to the changes in behaviour described in the stories reflects the knowledge translation approach we used in the home visits: not to tell people what to do, but rather to present them with evidence in a form that helps them to make decisions and act on them. The home visitors did not dictate what actions the households should take but rather showed what could happen with different courses of action; each household decided on actions that worked for their context. Socialising evidence for participatory action (SEPA) presents stakeholders with local evidence about actionable factors associated with an outcome, packaged to support decision-making [36, 37]. We have previously demonstrated a positive impact of SEPA as an intervention in different settings. Community discussions about local evidence of costs and benefits of childhood immunisation and non-immunisation led to a doubling of immunisation rates in Balochistan, Pakistan [40]. Community discussions about sexual health based on finding of an initial survey led to increased condom used in Canadian Indigenous communities [48]. Evidence-based community mobilization reduced dengue infection in Mexico and Nicaragua [49, 50].

Health literacy is the capacity to understand basic health information needed to make health decisions [51]. One effect of the repeated home visits to share and discuss evidence could have been to improve health literacy. There is evidence that health literacy is related to health outcomes. A study in India reported associations between maternal health literacy and child immunization, after taking account of multiple potential confounders, including maternal and paternal education, poverty, and availability of facilities; the authors noted that improvements in maternal health literacy could potentially be more rapidly achieved than changes in maternal education [52]. A recent systematic review of 22 studies of interventions to improve health literacy reported that 15 showed an improvement in at least one aspect of health literacy. Most of the included studies were from high income countries. The review authors suggested that health literacy interventions should consider using behaviour change models in their design and implementation [53]. Our use of the CASCADA model to analyse the stories of change related to home visits in this study is one such approach.

Intention to change is prominent in the Theory of Planned Behaviour, as the immediate precursor of action [38], and CASCADA is an adaptation of the Theory of Planned Behaviour. In stories from participants in an intervention for HIV infection prevention among young women in Southern Africa, *intention to change* was often evident, sometimes without actual change in behaviour [41]. In the present study, few storytellers mentioned an intention to change in their stories; rather they described the *actions* they had taken. This may be because we collected stories mainly from women and men who had been followed throughout a completed pregnancy, with opportunities to make changes during the pregnancy and after delivery. Some storytellers went further and described benefits they attributed to their actions, such as healthier children after immunization, or easier delivery after stopping heavy work during pregnancy. This demonstration that their actions worked increased the participants’ perceptions of the benefits of taking the action. According to the health belief model, this may mean they are more likely to continue taking the action in future [54].

The World Health Organization recommends increasing male involvement to improve maternal and newborn health [4]. There is good evidence that male involvement increases the uptake of maternal health services and improves health practices [21–23]. However, the impact on morbidity and mortality is less clear, and there are concerns that interventions to increase male involvement might even have negative consequences for gender equality [23, 55, 56]. Some of the stories from men in our study suggested profound changes. In some cases, the intervention seems to have been gender-transformative. In other cases, gender roles were unchanged, but men had improved knowledge and attitudes about what actions could be protective for the health of their wives and children [57]. We undertook a separate analysis focussing on the effect of the home visits on gender norms and dynamics [58].

The uniformly positive effect of the home visits intervention on men contrasts with our experience of a community mobilization intervention to reduce dengue transmission in Mexico and Nicaragua, where the positive impact was mainly among women and there was even a negative effect on some men [42]. The difference may be because the Bauchi home visits did not propose an increased role for women outside of their homes. None of the stories suggested that the visited men felt threatened or undermined by the visits to them or their wives.

### Stories from home visitors

Stories from the female and male home visitors highlighted positive changes in their lives that they attributed to their involvement in the work, similar to the benefits described by fieldworkers collecting data in South Africa [59]. Most women described economic empowerment because of their stipends for undertaking the work; male and female fieldworkers in a biomedical community-based research project in South Africa also reported financial benefits from their involvement in the project [60].

### Policy implications

Taken together with the quantitative evidence of impact of the home visits on maternal and child health outcomes [31–33], the narratives of change described here reinforce the idea that home visits to share evidence with pregnant women and their spouses can empower households to take action to improve their own situation. The Bauchi State Primary Health Care Development Agency has already committed to extending such home visits throughout the State, as funding permits.

### Limitations

Translation from Hausa to English may have lost some aspects of the stories. Field coordinators of the home visits program collected the stories, which might have constrained respondents from telling negative stories, even though the coordinators were not directly involved in undertaking or supervising the home visits. They advised story tellers they could tell about negative as well as positive changes, but nevertheless people overwhelmingly told positive stories. This is a recognised feature of the most significant change technique [61] and authors have recommended confirmation of reported benefits by other means [62]. In our case, we confirmed the impact of the home visits on knowledge, views and health outcomes using quantitative methods in the context of a cluster randomised controlled trial [31–33]. The changes described in the stories are likely to be context-specific and may not apply in other settings, but the approach of sharing evidence to allow households to take preventive actions is more widely relevant.

## Conclusions

The narratives of change gave important insights into likely mechanisms of impact of the home visits, at least in the Bauchi setting. The compatibility of our findings with the CASCADA results chain supports the use of this model in designing and analysing similar interventions in other settings. The indication that the home visits changed male engagement has broader relevance and contributes to the ongoing debate about how to increase male involvement in reproductive health.

## Data Availability

The datasets used and/or analysed during the current study are available from the corresponding author on reasonable request.

## Abbreviations

CASCADA: Conscious knowledge, attitudes, **s**ubjective norms, intention to change, discussion, action, agency
MMR: Maternal mortality ratio
LGA: Local government area
